# Image-guided surgery and novel intraoperative devices for enhanced visualisation in general and paediatric surgery: a review

**DOI:** 10.1515/iss-2021-0028

**Published:** 2022-02-02

**Authors:** Laura Privitera, Irene Paraboschi, Divyansh Dixit, Owen J Arthurs, Stefano Giuliani

**Affiliations:** Wellcome/EPSRC Centre for Interventional & Surgical Sciences, London, UK; Developmental Biology and Cancer Programme, UCL Great Ormond Street Institute of Child Health, London, UK; Faculty of Medicine, University of Southampton, Southampton, UK; Department of Clinical Radiology, NHS Foundation Trust, Great Ormond Street Hospital for Children, London, UK; NIHR GOSH Biomedical Research Centre, NHS Foundation Trust, UCL Great Ormond Street Institute of Child Health, London, UK; Department of Specialist Neonatal and Paediatric Surgery, NHS Foundation Trust, Great Ormond Street Hospital for Children, London, UK

**Keywords:** augmented reality, fluorescence-guided surgery, general surgery, image-guided surgery, intra-operative visualisation, novel devices, optical imaging, paediatric surgery

## Abstract

Fluorescence guided surgery, augmented reality, and intra-operative imaging devices are rapidly pervading the field of surgical interventions, equipping the surgeon with powerful tools capable of enhancing the surgical visualisation of anatomical normal and pathological structures. There is a wide range of possibilities in the adult population to use these novel technologies and devices in the guidance for surgical procedures and minimally invasive surgeries. Their applications and their use have also been increasingly growing in the field of paediatric surgery, where the detailed visualisation of small anatomical structures could reduce procedure time, minimising surgical complications and ultimately improve the outcome of surgery. This review aims to illustrate the mechanisms underlying these innovations and their main applications in the clinical setting.

## Introduction

The field of surgery faces an ever-increasing need for real-time intraoperative visualisation of small anatomical structures, such as vessels and nerves. In the last decade, there has been a great effort to develop novel technologies and devices that could better visualise vital organs, with the final aim to minimise surgical complications and improve outcomes. Technological innovations, such as fluorescence-guided surgery and augmented reality, are developing the broad field of image-guided surgery with the aid of more responsive and artificial intelligence enhanced robots [1], [2], [3]. In addition, innovative intra-operative imaging devices, such as intraoperative MRI, ultra-high frequency ultrasound, and photoacoustic imaging, are becoming crucial to give the surgeons novel tools for better field visualisation, anatomical prediction, and possible automated guidance [4], [5], [6], [7], [8], [9]. These novel imaging techniques and devices represent the future of surgery, and they are fast approaching clinical implementation. This review illustrates the most promising and innovative image-guided techniques and devices to enhance surgical visualisation of anatomical normal and pathological structures.

## Fluorescence-guided surgery (FGS)

FGS has proved to be a feasible tool for visualising vessels, organ perfusion, and tumours during surgical procedures thanks to generating a near-infrared (NIR) signal using different fluorescent markers [1, 2]. The main benefits of this novel technique are related to the absence of ionising radiations, the high contrast and sensitivity, and the good spatial resolution of fine anatomical structures, which could improve the real-time and high-resolution delineation of vital structures and tumours’ margins during surgery [2]. Both indocyanine green (IGC) and fluorescein sodium have been used in children and adults, with no significant reported side-effect [2]. In addition, a broad array of imaging and diagnostic technologies employ an experimental technique known as immunofluorescence consisting of fluorophore-labelled antibodies for specific and targeted visualisation.

### IGC and fluorescein sodium

ICG is a safe, anionic amphiphilic tricarbocyanine dye that received the Food and Drug Administration approval in the 1950s, and it is currently indicated for determining cardiac output, hepatic blood flow and performing ophthalmic angiography [1, 10]. Other promising but off-label applications include the visualisation of vascular anatomy [11], [12], [13], lymphatic vessels [14], [15], [16], biliary flow [17, 18], surgical margin definition during tumour resections [19, 20], bronchial tree visualisation [21] and ureter identification [22]. When intravenously administered, ICG is usually confined into the vascular stream by binding albumin, and it has hepatic clearance with an intravascular half-life of approximately 3 min [10].

Some of the most common clinical applications of ICG are linked to the visualisation of intestinal perfusion before anastomosis [11, 13] and the visualisation of blood and lymphatic vessels during laparoscopic urological surgery procedures [23, 24]. Moreover, the exclusive hepatic clearance leads to fluorescence cholangiography as an adjunct for laparoscopic cholecystectomy and Kasai hepatoportoenterostomy [17, 18]. The increased vascular permeability of the neo-angiogenetic vessels allows ICG to passively accumulate within the hepatic and non-hepatic primary tumour, enhancing margin delineation during surgical resections [19, 20, 25]. Other uses of ICG include its injection under CT guidance into pulmonary nodules to precisely localise neoplastic lesions intraoperatively [26]. In this regard, Quan et al. suggest that lung-specific inhalation delivery of ICG can be also helpful for the intraoperative visualisation of tumour margins in clinical practice [21]. Interestingly, when interstitially injected, ICG is protein-bound and confined into the lymphatic system. This can be exploited to track lymphatic drainage and to facilitate sentinel lymph node detection and biopsy [14], [15], [16, 27]. Apart from ICG, fluorescein sodium has also been described as a safe and inexpensive water-soluble dye to assess ischaemic bowels and intracranial tumours [28, 29].

### Fluorophore-labelled antibodies

Tumour-targeted fluorescent probes, such as fluorescently labelled monoclonal antibodies, are currently under investigation in adult oncology to detect viable tumour cells and better define surgical margins [30, 31]. Two of the most popular commercial near-infrared (NIR) cyanine heptamethine fluorophores for antibody conjugation are IRDye800CW and DyLight800.

There is only one clinical trial in the paediatric population, not yet recruiting, which will evaluate the safety, dosing and efficacy of Panitumumab-IRDye800 as an optical imaging agent in patients requiring brain surgery to remove tumours. For this clinical study, patients will undergo standard of care, histopathological-based, surgical resection of tumour 1–5 days after the infusion of the labelled antibody. IRDye800 is more commonly used in adult clinical trials, and we have summarised completed, terminated or currently recruiting studies using IRDye 800CW in the adult population (Table 1).

**Table 1:** Summary of clinical trials completed or active on molecular target fluorescent-guided surgery in the adult population.

Identifier	Title	Location	Drug	Status	Results
NCT02113202 [32]	Molecular fluorescence endoscopy in patients with familial adenomatous polyposis, using Bevacizumab-IRDye800CW	Netherlands	Bevacizumab-IRDye800CW	Completed	Near-infrared fluorescence molecular endoscopy is clinically feasible as a real-time, red-flag technique for detection of colorectal adenomas.
NCT01972373 [33]	Visualisation of rectal cancer during endoscopy, using a fluorescent tracer	Netherlands	Bevacizumab-IRDye800CW	Completed	Quantitative fluorescence endoscopy is a promising technique to aid clinical response assessment in patients with locally advanced rectal cancer and warrants further validation in larger clinical trials.
NCT01508572 [34]	VEGF-targeted fluorescent tracer imaging in breast cancer	Netherlands	Bevacizumab-IRDye800CW	Completed	Systemic administration of the Bevacizumab–IRDye800CW tracer is safe for breast cancer guidance and confirms tumour and tumour margin uptake.
NCT03913806 [35]	Fluoresence image guided surgery with a VEGF-targeted tracer in soft-tissue sarcomas in humans approach with Bevacizumab-IRDye 800CW	Netherlands	Bevacizumab-IRDye800CW	Completed	Fluorescence-guided surgery using 10 mg of Bevacizumab-800CW is feasible and safe for intraoperative imaging of soft-tissue sarcoma, allowing tumour detection and margin assessment intraoperatively.
NCT02736578 [36]	Cetuximab-IRDye 800CW and intraoperative imaging in finding pancreatic cancer in patients undergoing surgery	USA	Cetuximab-IRDye800	Terminated	First-in-human study to evaluate the use of multimodality molecular imaging in patients undergoing surgery for pancreatic cancer. The study proves the safety and feasibility of intraoperative, tumour-specific detection of PDAC using Cetuximab-IRDye800 with multimodal molecular imaging of the primary tumour and metastases was demonstrated.
NCT02129933 [37]	VEGF-targeted fluorescence near-infrared (NIR) endoscopy in (Pre)malignant oesophageal lesions	Netherlands	Bevacizumab-IRDye800CW	Completed	The concurrent use of VEGFA-guided NIR fluorescence molecular endoscopy and high-definition white-light endoscopy, following tracer administration, can be used to detect dysplastic and early cancerous lesions in patients with Barrett’s oesophagus.
NCT02855086 [38]	Cetuximab-IRDye 800CW in detecting tumors in patients with malignant glioma undergoing surgery	USA	Cetuximab-IRDye 800CW	Terminated	This first-in-human study demonstrates the feasibility and safety of antibody based imaging for contrast-enhancing glioblastomas.
NCT02743975	Near-infrared image guided surgery in pancreatic adenocarcinoma	Netherlands	Bevacizumab-800CW	Terminated	The study was terminated due to insufficient tumour-to-background ratios in the first three dose groups (4.5; 10; 25 mg).
NCT01987375 [39]	Cetuximab IRDye800 study as an optical imaging agent to detect cancer during surgical procedures	USA	Cetuximab-IRDye800	Terminated	Commercially available antibodies can be fluorescently labelled and safely administered to humans, potentially improving outcomes in clinical oncology.
NCT03134846 [40]	Image guided surgery for margin assessment of head and neck cancer using Cetuximab-IRDye800CW conjugate	Netherlands	Cetuximab-IRDye800CW	Recruiting	Authors were able to use a lower dose Cetuximab-800CW than previously described, while remaining a high sensitivity for tumour detection due to application of equipment optimised for IRDye800CW detection.
NCT04459065 [41]	Evaluation of IRDye800CW-nimotuzumab in lung cancer surgery	Canada	IRDye800CW-nimotuzumab	Recruiting	Preliminary results on pre-clinical studies demonstrate that nimotuzumab conjugated to IRDye800CW is safe and does not exhibit toxicities commonly associated with EGFR targeting antibodies.
NCT03925285 [42]	Image guided surgery in sinonasal inverted papilloma	Netherlands	Bevacizumab-800CW	Recruiting	Preliminary results show that a fluorescence grid analysis could serve as a valid method to evaluate fluorescence molecular imaging in piecemeal surgeries.

Even if the past decade has undoubtedly witnessed significant advances in the clinical application and technical development of fluorescent optical imaging, there is the need to translate these technologies more stably and effectively. Further studies involving larger cohorts of patients and continued experience with fluorophores and optical imaging systems will soon allow FGS to become a well-establish technique to improve surgical outcomes in surgery.

## New intra-operative devices

### Augmented reality (AR)

**Figure 1:**
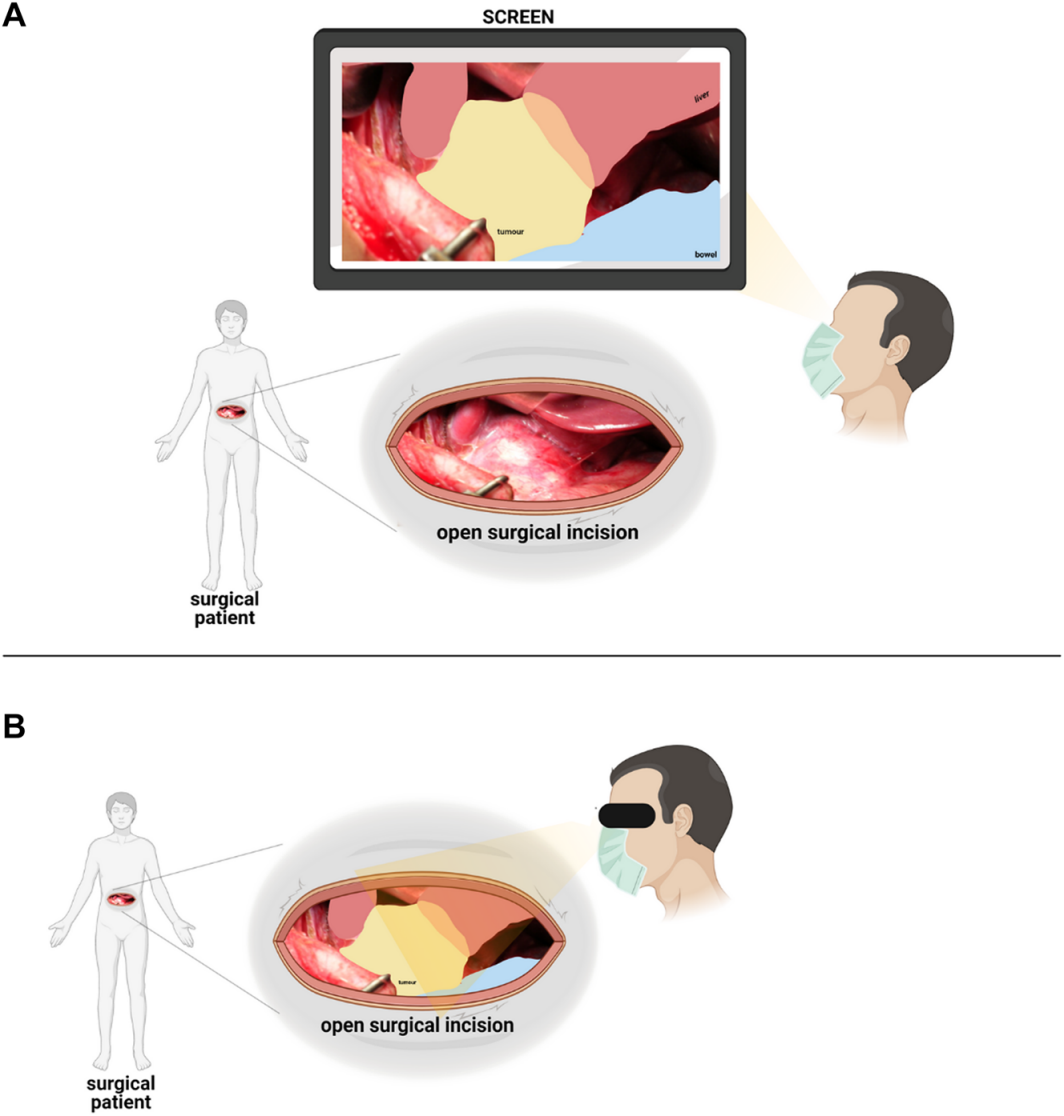
AR in the surgical field through the visualisation of data projected on a screen (panel A) or with a head-mounted display (black arrow in panel B) that superimposed objects onto real-time images (panel B). The picture shows a tumour (yellow) before surgical resection. The area in orange shows the extension of the tumour into the liver (red). The bowel is marked in light blue.

Augmenting pre-operative imaging into a 3D model superimposed onto a real-time surgical field represents an exciting novel opportunity to enhance surgical practice. AR has the potential to act as an effective intraoperative adjunct by increasing information accessible to a surgeon whilst remaining aseptic and within anaesthetic-implicated time restraints [43]. Augmented reality technology can be defined into clear stages: the acquisition of 2D pre-operative images to produce a 3D model of patient-anatomy, then calibrating the model onto the real-time field so that changes in view angles are simultaneously adjusted in the 3D model. The modality by which the surgeon views the artificial image overlay can vary and includes artificial colouring/texturing, enhancing tissue identification and overall anatomical depiction (Figure 1) [2, 3]. Despite the potential benefit of AR, there are significant limitations to its use. AR is an expensive technology to use, requiring relatively powerful microcomputers balanced with the need for equipment to be practical to use. In addition, the differences between pre-operative imaging and actual intraoperative anatomy may be significant and, reducing the AR reliability and usability during surgery. This is especially true in the paediatric population, where minor errors in mapping imaging to AR visualisation can lead to major inconsistencies [44].

AR applications in adult surgery have been extensive, especially in neurosurgery, otolaryngology and orthopaedics, due to spatial preservation of internal structures with anatomical landmarks intraoperatively [45, 46]. On the other hand, the intraoperative use of AR in paediatric surgery is still limited**.** Nonetheless, the use of AR has been explored in the field of endoscopic surgery. Ieiri et al. discussed six paediatric cases undergoing laparoscopic splenectomy, where pre-operative 3D imaging produced corresponded to body surface markers intraoperatively to produce an augmented reality. The real strength outlined was of hidden structures that would not be visualised without further exploration. This may be a particular strength in a paediatric setting due to a higher prevalence of “anomalous anatomy”, whereby identifying unique anatomy could reduce navigation time, improve accuracy and ultimately improve the success of surgery [44]. Souzaki et al. provided some valuable insights into further potential benefits of this technology used intraoperatively during the tumour resection without complications. They highlighted the particular benefit of AR with malignancies, as tissue discrimination can be difficult due to peri-organ adhesions formed from neoadjuvant therapy and re-operative factors [47]. Finally, Pennacchietti et al. described intraoperative AR in a neuronavigation setting, which may be easier to implement with more fixed spatial anatomical relationships in head and neck surgery than abdominal surgery. Despite the difficulty in interpreting the results due to the lack of controls for comparison of patient outcomes, the authors stress the accuracy of the AR system intraoperatively, especially in identifying anatomical landmarks to better execute the surgical plan and its reproducibility [48].

### Intraoperative contrast-enhanced ultrasound (CEUS)

**Figure 2:**
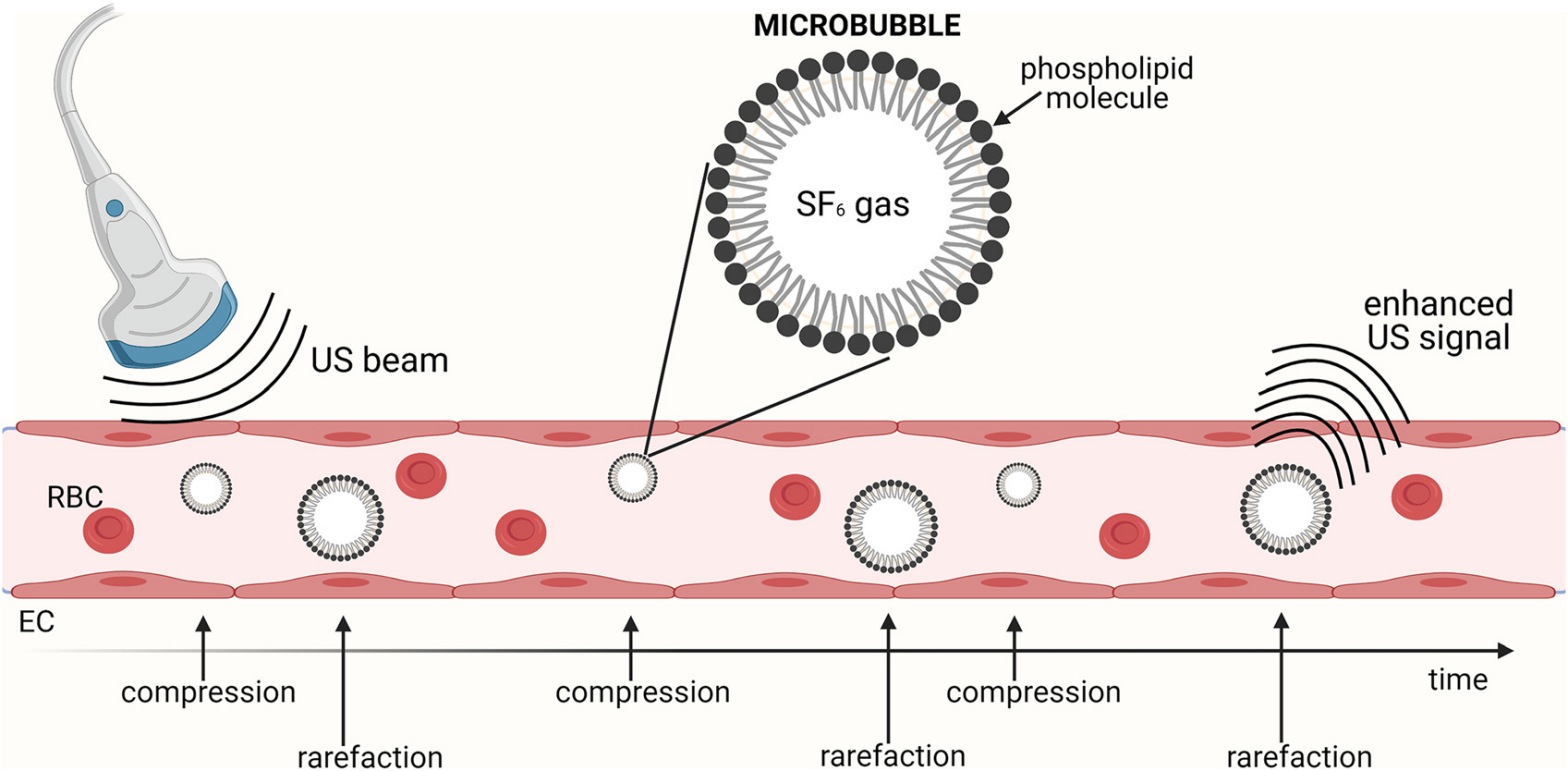
Schematic representation of contrast-enhanced ultrasound mechanism of action, with intravenously administered contrast agent. Ultrasound contrast agents consist of gas-filled microbubbles (1–10 µm) with a lipid, protein, or polymer shell. The pressure changes induced by the ultrasonic waves lead microbubbles to contract (*compression*) and expand (*rarefaction*) to a higher degree compared to the surrounding tissues. This, along with the impedance mismatch between the microbubble and surrounding fluid caused by the gas, makes the bubbles highly echogenic. **Abbreviations:** RBC, red blood cell; EC, endothelial cell.

CEUS is an attractive imaging modality because of its high safety profile, lack of ionising radiation, absence of specific patient preparation or sedation, and the possibility of bedside access (Figure 2). Its use is well established in many clinical settings for the adult population, with detailed guidelines on its application [49, 50]. Ultrasound contrast agents can be administered intravenously to characterise the enhancement patterns of focal lesions; into the urinary bladder for detection and grading of vesicoureteral reflux; or endo-cavitary for drainage or detection of an abnormal communication between two cavities [51, 52].

The use of CEUS in the paediatric population has been increasingly growing for a wide variety of indications, providing stimulating improvements to diagnosis, treatment and intraoperative surgical management. For example, CEUS appeared to have a great potential for the accurate visualisation, characterisation and malignancy assessment of hepatic tumours at the time of resection [53]. Arita et al. further validated its utility in the definition of hepatic lesions as their results showed a strong correlation between CEUS parameters and the histological features of the hepatocellular carcinomas [54]. The contrast-enhanced US may be able to improve on the performance of conventional B_mode ultrasound, where there may be a discrepancy between preoperative and intraoperative findings in a significant minority of children. For example, Felsted found that 20% of intraoperative US gave discordant results compared to pre-operative MRI, including the extent of tumour involvement and diffuse vs. focal disease. In 3/19 cases the operative plan was altered [55]. CEUS has also been used in neurosurgery to assist surgeons in distinguishing margins between viable intracranial tumours and adjacent healthy parenchyma [56].

Not only CEUS can help guide tumours’ biopsy to improve the accuracy of the final diagnosis, but the real-time visualisation of the vascular pattern in higher-grade tumours could also improve the intraoperative management by identifying structures at high risk for bleeding or the need for more aggressive margins at the time of resection [57]. Prantl et al. explored another application into the intraoperative field by using CEUS to assess the viability of the femoral head before and after developmental dysplasia of the hip reduction [58]. Finally, the use of CEUS in urology is mainly related to evaluating vesicoureteral reflux’s treatment, where the real-time intraoperative assessment of residual reflux following the injection of endoscopic bulking agents allows for repeated injections to improve the success of the procedure [59].

### Ultra-high frequency ultrasound (UHFUS)

In the last decade, novel matrix transducers able to produce ultra-high frequency emission have been developed, enabling higher spatial resolution and improvement of imaging quality of superficial layers, at the expense of a shallower US beam penetration (23.5 mm when applying 48 MHz frequencies vs. 10 mm using 70 MHz frequencies) [60, 61]. Despite the lack of consensus around the cut-off frequencies for very high and ultra-high frequencies, UHFUS can be defined as a diagnostic technique characterised by the use of frequencies ranging from 30 to 100 MHz [62], [63], [64]. The highly detailed image resolution and its ability to provide a thorough investigation of small anatomy favoured the first clinical applications of UHFUS, which predominantly involved the evaluation of the skin and vessels [64]. Regarding its safety, pre-clinical research on mice demonstrated the absence of significant biological effects related to the increase in thermal and mechanical energy deposition in tissues [65].

In the adult population, UHFUS is expanding rapidly in different fields, including dermatological applications [66, 67], vascular analysis [68, 69], lymphatic channel identification [70], and musculoskeletal evaluation of the hand anatomy for presurgical planning [71, 72].

The use of UHFUS in children is still in very early development but is appealing for smaller anatomy without using radiation. One of the most common applications addresses difficult vascular access, with UHFUS being a promising tool in reducing vascular injuries related to peripheral arteries cannulation and central venous access [6]. Ultrasonography as a means of examining soft tissue was also explored by Granéli et al., whose hypothesis was that UHFUS could be used to differentiate aganglionic and ganglionic bowel wall during surgery for Hirschsprung Disease [7]. In fact, there is no other intraoperative method other than frozen biopsy to secure the level of ganglionic bowel. In their study, they used 70 MHz transducers on a total of 11 unique bowel segment samples. Findings at the ultrasonography were confirmed by histo-immunologic analysis in 42 out of the 44 analysed sites, proving the potential use of UHFUS intra-operatively for instantaneous verification of aganglionosis and a more precise bowel length resection [7].

### Intraoperative MRI (iMRI)

Intraoperative MRI has been particularly beneficial in neurosurgery, where it provides the evaluation of surgical execution by delineating the relationships with surrounding functionally relevant structures and assessing the dynamic changes (i.e. brain shift) that occur during surgery in near real-time. This is important particularly in children, where the extent of tumour removal represents the main prognostic factor in malignant intracranial tumours [4]. Giordano et al. looked at the safety, advantages, and limitations of iMRI for neurosurgical procedures in paediatric patients showing that, particularly in low-grade gliomas and craniopharyngiomas, iMRI was most effective in increasing the extent of tumour resection [4]. Wach et al., in their metanalysis, evaluated the impact of iMRI on surgery of paediatric brain tumours by analysing data on the frequency of histopathologic entities, additional resections secondary to iMRI, rate of gross total resections in glioma surgery, and neurologic outcome after surgery. Overall, iMRI-guided surgery seems to improve paediatric glioma surgery, with a frequency of new neurologic deficits after iMRI-guided surgery within the normal range of paediatric neuro-oncologic surgery [73]. About brain tumour resection, Avula et al. compared the effectiveness of the final intraoperative MRI and early postoperative MRI as baseline scans to evaluate whether the former could be used as a postoperative baseline. Their results showed no difference between iMRI and postoperative scans in identifying residual tumour when standard imaging guidelines are followed and the evaluation is done jointly by the operating neurosurgeon and neuroradiologist [74]. Gallieni et al. published their experience in treating six children with minimally pneumatised sphenoid sinus, demonstrating that the transsphenoidal approach is still possible with a minimal level of pneumatisation, especially with the support of neuronavigation and iMRI. They reported no perioperative complications and no mortality cases due to the surgical approach, demonstrating that the transsphenoidal approach can be safely used even in minimally pneumatised sphenoid sinus as in young children [75].

Another application of iMRI was reported by Di Carlo et al., who determined the safety and efficacy of iMRI guided surgical reconstruction to identify the pelvic floor anatomy during the closure of classic bladder exstrophy and cloacal exstrophy. The intraoperative registration was performed after pre-operative planning with a paediatric radiologist using five anatomical landmarks immediately before surgery initiation. There was 100% accuracy in all patients in the correlation of gross anatomical landmarks with 3D iMRI identified landmarks, and all patients had successful closure without any major complications. Thus, intraoperative MRI represents an effective way to accurately identify pelvic anatomy and offers a unique surgical skill education opportunity in this complex reconstructive operation [5].

Finally, Jarboe et al. demonstrated that muscle-sparing laparoscopic anorectoplasty using real-time MRI is feasible and facilitates needle placement through the sphincter muscle complex when repairing imperforate anus [76]. In fact, a challenge when performing this procedure is to put the neo-rectum into the centre of the sphincter muscle complex with limited muscle injury and scarring. The authors treated five children using real-time MRI to delineate the complex and guide the needle through the centre. After needle placement, laparoscopic mobilisation, fistula takedown and pullthrough were performed using the needle to guide dilations to create a tract into the pelvic floor. They reported no intraoperative complications, although one patient had temporary urinary retention postoperatively.

### Photoacoustic imaging (PAI)

**Figure 3:**
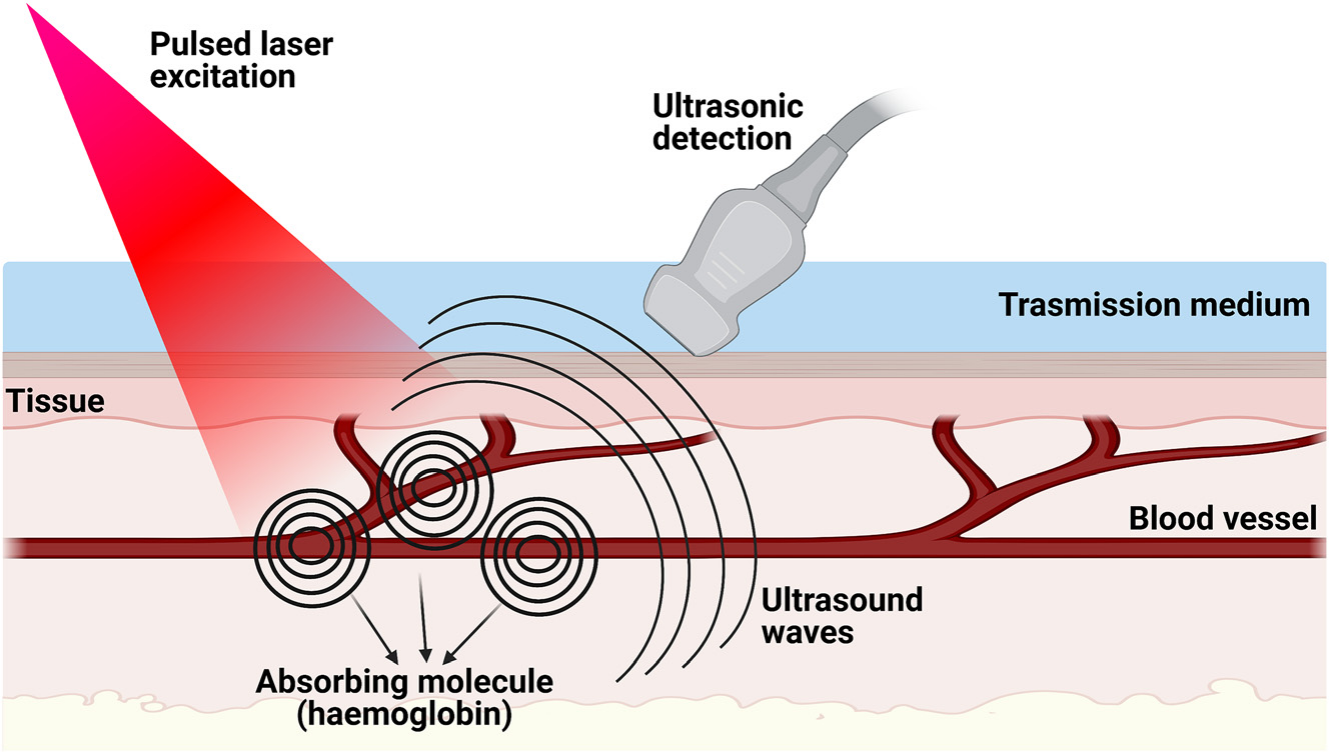
Schematic representation of photoacoustic imaging mechanism of action. The absorption of light by endogenous chromophores (or pigments) generates heat, leading to a pressure change. The resulting fleeting expansions generate an ultrasound wave which can then be detected and used to produce clear, high-resolution images of biological structures.

PAI is an emerging non-invasive technique imaging modality with great potential in the clinical field and the operating room as a standard technological assistant. Its functioning is based on the absorption of short, low-energy non-ionising laser pulses of specific wavelengths by light-absorbing molecules of the imaged tissue (i.e. haemoglobin, melanin, water and lipids) [77]. The NIR spectral range (600–900 nm) offers the greatest penetration depth extending to several centimetres, making PAI particularly well suited to visualising the vasculature without using contrast agents, with scalable spatial resolution ranging from tens to hundreds of micrometres. The arrival of these waves at the tissue surface leads to an initial pressure increase, which then relaxes and results in the emission of broadband low-amplitude acoustic waves. Ultrasonic transducers detect the generated waves, and the image is then reconstructed, knowing the speed of the sound, by measuring the time of arrival of the acoustic waver (Figure 3) [78].

There is a wide range of possibilities regarding PAI guidance for surgical procedures and minimally invasive surgeries in the adult population. Augmented surgical tools can be created by attaching the optical fibres to the surgical instruments, and the photoacoustic signals can be detected with an ultrasound probe. Photoacoustic imaging has been used in liver surgeries to determine the location of a major hepatic blood vessel based on its appearance as a focused signal rather than a diffuse signal, which is predominant in the liver tissue [79]. The utility of PAI has also been proved in spinal fusion surgeries, performed to alleviate pain or neurologic deficit, and in the gynaecological field, where iatrogenic injuries to the ureter are often caused by clamping, clipping, or cauterising the uterine arteries [80, 81]. Allard M et al. performed a feasibility study to use PAI to visualise both the ureter and uterine artery during hysterectomies performed with the Da Vinci^®^ robot. Their experiments were performed in a mock operating room, and their results proved that photoacoustic imaging is a promising approach to enable visualisation of the uterine arteries to guide hysterectomies and be effectively integrated into robotic systems [82].

Children and adolescents might be good candidates for this scanner as their organs and muscles are closer to the surface. In fact, the image quality can be significantly influenced by air, thick bones, body fat, and body hair. Interventional PAI could be valuable for minimally invasive foetal surgery by visualising superficial and subsurface chorionic placental vasculature. In the study by Maneas et al., the authors imaged a normal placenta and a placenta from an identical twin pregnancy complicated by twin-to-twin transfusion syndrome, which was treated with photocoagulation *in utero*, providing the first demonstration that 3D photoacoustic imaging of the human placenta can generate detailed maps of surface and subsurface vasculature to a depth of approximately 7 mm. Superficial chorionic placental vessels were visualised, while fetoscopy photocoagulation was manifested as an absence of signal (Table 2) [8].

**Table 2:** Summary of principle of mechanism, advantages and limitations of the investigated intra-operative devices.

Intra-operative devices	Principle	Advantages	Limitations
Augmented reality (AR)	Superimposition of pre-operative images onto the surgical field	– Real-time enhancement of the surgical procedure – Better appreciation of 3D structures – Possibility of tactile feedback – Future educational advancement	– High costs – Potential registration errors – Lack of devices – Protection of personal identifiable data
Intraoperative contrast-enhanced ultrasound (CEUS)	CEUS combines ultrasound imaging with intravenous contrast to improve the visualisation of blood vessels and organs.	– Non-invasive and non-ionising – Real-time detection – May offer improved diagnostic performance than conventional ultrasound – Safety profile of contrast agents – Absence of specific patient preparation/sedation – Possibility of bedside access	– Small risk of an allergic reaction – Instability of contrast agents – Operator dependent – Location of the lesion and patients’ characteristics affect images acquisition – Limited sonic window and scattering of the sound waves due to anatomical changes – Requires experience and training
Ultra-high frequency ultrasound (UHFUS)	Novel matrix ultrasound transducers with frequencies ranging from 30 to 100 MHz	– Non-invasive and non-ionising – Real-time detection – Higher spatial resolution – Better quality of images from superficial layers – Absence of specific patient preparation/sedation	– Reduced penetration depth – Image acquisition can be affected by air – Operator dependent – Requires experience and training Higher costs
Intraoperative magnetic resonance imaging (MRI)	Magnetic resonance imaging obtained during surgery	– High-resolution images – Real-time guidance during surgery – Allows surgeons to perform safer and more effective surgery of some tumours	– High cost of MRI systems – Side effects to contrast agents – Need to prevent hypothermia during long procedures – Limited operative positioning – Specific equipment requirement
Photoacoustic imaging (PAI)	Non-invasive technique based on the absorption of non-ionising laser pulser by endogenous light-absorbing molecules	– Non-invasive – Near real-time imaging capability – Relies mainly on endogenous contrast – High-quality 3D anatomical images – Greater specificity than ultrasound imaging – Can provide functional information	– Penetration depth is limited by optical and acoustic attenuations – Need for highly sensitive suitably broadband receivers – Lack of suitable laser systems

## Conclusions

Fluorescence-guided surgery, augmented reality, iMRI, UHFUS, and PAI represent an exciting novel opportunity to enhance surgical practice, with the potential to yield significant advantages in specific challenges faced with paediatric patients. Further testing of these novel intraoperative techniques together with the use of robotic instruments will determine their role in surgical precision and visualisation, which should lead to highly precise resection of targeted tissues, revolutionising surgery.

## Supplementary Material

Supplementary Material
