# Supplementary material for: Image-guided surgery and novel intraoperative devices for enhanced visualisation in general and paediatric surgery: a review
Source: Innov Surg Sci. 2022 Feb 2;6(4):161–72. doi: 10.1515/iss-2021-0028 (PMC9294338; doi:10.1515/iss-2021-0028)
Supplement: Supplementary file 1 — Supplementary Material [file j_iss-2021-0028_suppl.pdf]

Reviewer Assessment

L. Privitera et al.: Image-guided surgery and novel inoperative devices for enhanced visualisation in general and paediatric surgery: A review

Reviewers' Comments to Original Submission

Reviewer 1: Munsterer, Oliver

Date received: 02-Sep-2021
Reviewer recommendation: **Return to author for major modifications**
Reviewer overall scoring: **High**

Assessment Form scores: 5 = High/Yes; 3 = Medium/Adequate; 1 = Low

Is the subject area appropriate for the journal	5				
Does the title clearly reflect the paper's content?		4			
Does the abstract clearly reflect the paper's content		4			
Do the keywords clearly reflect the paper's content?		4			
Does the introduction present the problem clearly?		4			
Are the results/ conclusions justified?		4			
How comprehensive and up-to-date is the subject matter presented?	5				
How adequate is the data presentation?		4			
Are units and terminology used correctly?	5				
Is the number of cases adequate?	5				
Are the experimental methods/ clinical studies adequate?	5				
Is the length appropriate in relation to the content?			3		
Does the reader get new insights from the article?	5				
Please rate the practical significance.	5				
Please rate the accuracy of methods.	5				
Please rate the statistical evaluation and quality control.				2	
Please rate the appropriateness of the figures and tables.	5				
Please rate the appropriateness of the references.					
Please evaluate the writing style and use of language.					
Please judge the overall scientific quality of the manuscript.					
Are the methods used worthy of reproduction in greater deal?	yes				
Would you be willing to review a revision of this manuscript?	yes				

Comments to author: This is a review of novel and innovative techniques for intraoperative enhanced imaging and image-guidance in general and paediatric surgery. This is a timely topic and the authors give a narrative up-to-date report of recently

introduced technology. In my opinion, this article is informative, but I have some suggestions for improvement. before I can recommend publication:

1 - Title: I was confused about the "color-coded" term in the title. While I understand the intention, some of the techniques actually don't use color, and it probably implies a simplicity that is unwarranted. On the other hand, I think that the term "Review" is very important in the title because it describes the article and makes it better searchable. So perhaps "A comprehensive review of novel devices ..." would be a more appropriate title.

2 - Style: The authors have chosen a very lengthy narrative style to present these interesting innovations. The reader learns what is out there and what is possible, but the long text somewhat overrides the science on outcome and usability studies. I would suggest shortening the text and referring on a concise overview table or graph for each discussed method.

3 - Table: the table (Table 1) is a lengthy list of studies that have been performed, but it does not mention outcome. To me as a reader, I am not so much interested in how many studies are underway or recruiting but more on the objective data on benefits (and/or risks) of each method. Again, I would suggest a table for each of the techniques that lists peer-reviewed, published studies that evaluated the individual method (perhaps adult/pediatric studies can be marked somehow in the table).

4 - Cost: I know that cost is sometimes hard to calculate because it depends on setting, brand used etc. but it would be great to have a ballpark number on what should be expected in terms of resources and funds necessary to implement those new techniques..

Reviewer 2: anonymous

Date received: 18-Oct-2021

Reviewer recommendation: **Return to author for minor modifications**

Reviewer overall scoring: **Excellent**

Assessment Form scores: 5 = High/Yes; 3 = Medium/Adequate; 1 = Low

Is the subject area appropriate for the journal	5				
Does the title clearly reflect the paper's content?	5				
Does the abstract clearly reflect the paper's content		4			
Do the keywords clearly reflect the paper's content?	5				
Does the introduction present the problem clearly?	5				
Are the results/ conclusions justified?	5				
How comprehensive and up-to-date is the subject matter presented?	5				
How adequate is the data presentation?	5				
Are units and terminology used correctly?	5				
Is the number of cases adequate?	5				
Are the experimental methods/ clinical studies adequate?		4			
Is the length appropriate in relation to the content?		4			
Does the reader get new insights from the article?	5				
Please rate the practical significance.	5				
Please rate the accuracy of methods.	5				
Please rate the statistical evaluation and quality control.	5				
Please rate the appropriateness of the figures and tables.		4			
Please rate the appropriateness of the references.	5				
Please evaluate the writing style and use of language.	5				

Please judge the overall scientific quality of the manuscript.	5				
Are the methods used worthy of reproduction in greater deal?	Yes				
Would you be willing to review a revision of this manuscript?	Yes				

Comments to author: This is a very well designed and written review on colour coded surgery and novel devices for enhanced intraoperative visualisation.
The review covers all modern approaches in the scientific and clinical field.
The only thing, I would like to ask is a table giving an overview on the properties of the mentioned new technologies..

Authors' Response to Reviewer Comments

Date received: 11-Nov-2021

Response to reviewer 1

This is a review of novel and innovative techniques for intraoperative enhanced imaging and image-guidance in general and pediatric surgery. This is a timely topic and the authors give a narrative up-to-date report of recently introduced technology. In my opinion, this article is informative, but I have some suggestions for improvement before I can recommend publication:

Q1 Title: I was confused about the "color-coded" term in the title. While I understand the intention, some of the techniques actually don't use color, and it probably implies a simplicity that is unwarranted. On the other hand, I think that the term "Review" is very important in the title because it describes the article and makes it better searchable. So perhaps "A comprehensive review of novel devices ..." would be a more appropriate title.

R1 Thank you for your feedback. We agree that the term "colour-coded" might be misleading and therefore we have changed it to "image-guided" surgery. The two main topics of the manuscript are fluorescence guided surgery and novel intraoperative devices. We agree that the addition of the term "review" helps to explain the purpose of the manuscript and therefore has been added.

Q2 Style: The authors have chosen a very lengthy narrative style to present these interesting innovations. The reader learns what is out there and what is possible, but the long text somewhat overrides the science on outcome and usability studies. I would suggest shortening the text and referring on a concise overview table or graph for each discussed method.

R2 Thank you for your comment. We have structured our paragraphs in such a way where we provide a short technical introduction followed by a series of examples present in the clinical literature. We believe that this will offer the reader an immediate source of information for them to explore further should they wish. We have also added a table at the end of the manuscript summarising principles, advantages and limitations of these techniques in order to provide the reader with a quick overview.

Q3 Table: the table (Table 1) is a lenghty list of studies that have been performed, but it does not mention outcome. To me as a reader, I am not so much interested in how many studies are underway or recruiting but more on the objective data on benefits (and/or risks) of each method. And, I would suggest a table for each of the techniques that lists peer-reviewed, published studies that evaluated the individual method (perhaps adult/pediatric studies can be marked somehow in the table).

R3 Thank you for your feedback regarding Table I. We have now changed the table accordingly to your advice. With regards to the addition of multiple tables listing published-studies, as per Q2 we believe that our narrative style provides the reader with a more straightforward insight on these innovations.

Q4 Cost: I know that cost is sometimes hard to calculate because it depends on setting, brand used etc. but it would be great to have a ballpark number on what should be expected in terms of resources and funds necessary to implement those new techniques.

R4 Thanks for this comment. Although we understand the importance of costings in these new technologies, we haven't added a cost section to the manuscript. The first reason is that costing of these technologies are not commonly reported in the published paper and we are not experts in calculating them. The second reason is that we believe it is out of the scope of the review to provide the costings as it will require skills and methodology that we do not have in the team.

Response to reviewer 2

Q1 This is a very well designed and written review on colour coded surgery and novel devices for enhanced intraoperative visualisation. The review covers all modern approaches in the scientific and clinical field. The only thing, I would like to ask is a table giving an overview on the properties of the mentioned new technologies.

R1 Thank you for your feedback. As suggested, we have now added a table summarising the principles, advantages and limitations of the intraoperative devices mentioned in the manuscript.

Comment by the Guest Editor of the Thematic Issue on the revised version

All reviewer comments were addressed adequately and the manuscript should be published in its present stage.
